# Psychiatry resident physician duty hours, resting times and European Working Time Directive compliance in Spain

**DOI:** 10.1192/j.eurpsy.2024.809

**Published:** 2024-08-27

**Authors:** J. P. Carrasco Picazo, D. A. Sánchez Martínez, P. Estrella Porter, R. Ruiz-Montero, A. H. Aginagalde Llorente, E. García-Camacho, J. Navarro, A. Cerame del Campo

**Affiliations:** ^1^Psychiatry, Hospital Provincial de Castellón, Castellón; ^2^Oncology, IMIB-Arrixaca , Murcia; ^3^Preventive Medicine, Hospital Clínico Universitario de Valencia, Valencia; ^4^Maimonides Biomedical Research Institute of Cordoba (IMIBIC), Córdoba; ^5^Sub Directorate for Public Health and Addictions of Gipuzkoa, Ministry of Health of the Basque Government, San Sebastian; ^6^Cardiology, Complejo Hospitalario Universitario Toledo, Toledo; ^7^Intensive Care, Hospital Universitario Virgen del Rocío, Sevilla; ^8^Psychiatry, Plan de Atención Integral al Profesional Sanitario Enfermo, Madrid, Spain

## Abstract

**Introduction:**

There is a growing interest in understanding the impact of duty hours and resting times on training outcomes and the well-being of resident physicians. Psychiatry resident’s duty hours in Spain comprise a regular working schedule of 37.5h per week and a minimum of 4 mandatory on-call shifts. The most recent duty hours regulations in Spain were transposed from the European Working Time Directive (EWTD). According to Spanish Law, doctors cannot work for more than 48h per week and need to have resting times per day (at least 12h), per week (at least 36h) as well as annual leave (at least a month). However, there is practically no data on this situation in psychiatry resident physicians.

**Objectives:**

Our aim is firstly, to describe the number of shifts performed by psychiatry resident physicians in Spain. Secondly, to describe compliance with the daily and weekly rests compared to those set in national and European law. Finally, to analyse the difference by demographic variables (gender and year of residency), in both the number of on-call duty shifts and compliance with rests.

**Methods:**

A descriptive cross-sectional study was designed through an online survey adapted from the existing literature. The target population were Spanish psychiatry resident physicians undergoing PGT who started their specialist training during the years 2018–2021. The survey was disseminated through the Spanish regional medical councils to all active psychiatry resident physicians by mail as well as through informal communication channels. The study was authorised by the Spanish Medical Organization’s General Assembly which is the highest ethical and deontological body of physicians in Spain.

**Results:**

55 responses were obtained, of which 61.82% identified as females. The mean number of on-call shifts in the last 3 months was 14.05. This mean was highest in women 14,32 and in the cohort of 2020 15.46 (first year of residency). Among the resident physicians surveyed, 66.07% exceeded the 48h per week limit set by the EWTD and 7% of them did not rest after a 24-h on-call shift. Furthermore, 22% of respondents did not have a day-off after a Saturday on-call shift. The mean working hours when not resting after an on-call-shift were 7 hours. The comparison by gender and year of residency of the main variables can be seen in figures 1 and 2 respectively.

**Image:**

**Figure:**
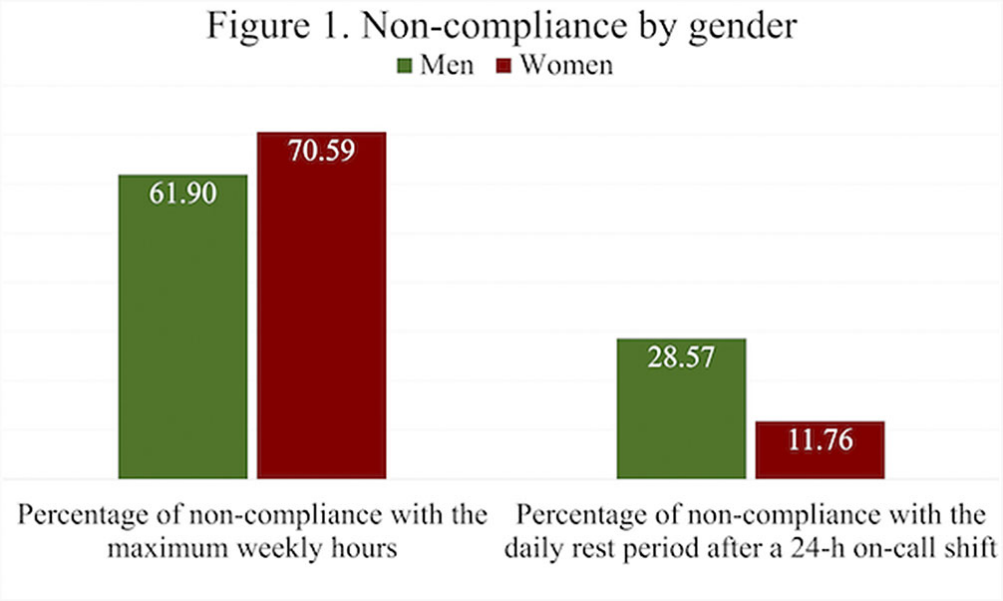

**Image 2:**

**Figure:**
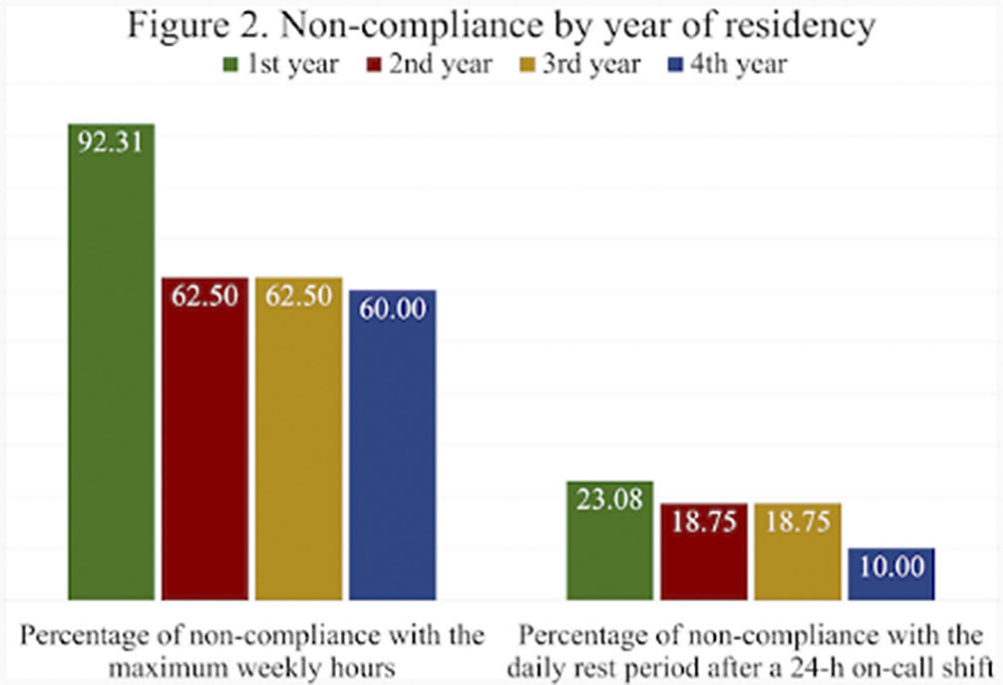

**Conclusions:**

Psychiatry resident physicians in Spain greatly exceed the established 48 h/week EWTD limit. Likewise, non-compliance with labour regulations regarding mandatory rest after on-call duty and minimum weekly rest periods are observed. Differences can be seen by gender and year of residency. The situation described could potentially create a high-risk situation for the health and psychosocial well-being of resident physicians, hinder learning outcomes and could lead to suboptimal patient care.

**Disclosure of Interest:**

None Declared

